# The Impact of the Availability of Immunotherapy on Patterns of Care in Stage III NSCLC: A Dutch Multicenter Analysis

**DOI:** 10.1016/j.jtocrr.2021.100195

**Published:** 2021-06-06

**Authors:** Merle I. Ronden, Idris Bahce, Niels J.M. Claessens, Nicole Barlo, Max R. Dahele, Johannes M.A. Daniels, Caroline Tissing-Tan, Edo Hekma, Sayed M.S. Hashemi, Antoinet van der Wel, Femke O.B. Spoelstra, Wilko F.A. R. Verbakel, Marian A. Tiemessen, Marjolein van Laren, Annemarie Becker, Svitlana Tarasevych, Cornelis J.A. Haasbeek, Karen Maassen van den Brink, Chris Dickhoff, Suresh Senan

**Affiliations:** a Department of Radiation Oncology, Amsterdam UMC, Amsterdam, the Netherlands; b Department of Pulmonology, Amsterdam UMC, Amsterdam, the Netherlands; c Department of Pulmonology, Rijnstate Ziekenhuis, Arnhem, the Netherlands; d Department of Pulmonology, Noordwest Ziekenhuisgroep, Alkmaar, the Netherlands; e Radiotherapy Group, Institute for Radiation Oncology, Arnhem, the Netherlands; f Department of Surgery, Rijnstate Ziekenhuis, Arnhem, the Netherlands; g Department of Pulmonology, Dijklander Ziekenhuis, Hoorn & Purmerend, the Netherlands; h Department of Pulmonology, Zaans Medisch Centrum, Zaandam, the Netherlands; i Department of Surgery, Amsterdam UMC, Amsterdam, the Netherlands

**Keywords:** Non–small cell lung cancer (NSCLC), Stage III, Multidisciplinary tumor board (MDT), Patterns of care, Immunotherapy

## Abstract

**Introduction:**

Treatment patterns in stage III NSCLC can vary considerably between countries. The PACIFIC trial reported improvements in progression-free and overall survival with adjuvant durvalumab after concurrent chemoradiotherapy (CCRT). We studied treatment decision-making by three Dutch regional thoracic multidisciplinary tumor boards between 2015 and 2019, to identify changes in practice when adjuvant durvalumab became available.

**Methods:**

Details of patients presenting with stage III NSCLC were retrospectively collected. Both CCRT and multimodality schemes incorporating planned surgery were defined as being radical-intent treatment (RIT).

**Results:**

Of 855 eligible patients, most (95%) were discussed at a thoracic multidisciplinary tumor board, which recommended a RIT in 63% (n = 510). Only 52% (n = 424) of the patients finally received a RIT. Predictors for not recommending RIT were age greater than or equal to 70 years, WHO performance score greater than or equal to 2, Charlson comorbidity index greater than or equal to 2 (excluding age), forced expiratory volume in 1 second less than 80% of predicted value, N3 disease, and period of diagnosis. Between 2015 to 2017 and 2018 to 2019, the proportion of patients undergoing CCRT increased from 34% to 42% (*p* = 0.02) and use of sequential chemoradiotherapy declined (21%–16%, *p* = 0.05). Rates of early toxicity and 1-year mortality were comparable for both periods. After 2018, 57% of the patients who underwent CCRT (90 of 159) received adjuvant durvalumab.

**Conclusions:**

After publication of the PACIFIC trial, a significant increase was observed in the use of CCRT for patients with stage III NSCLC with rates of early toxicity and mortality being unchanged. Since 2018, 57% of the patients undergoing CCRT went on to receive adjuvant durvalumab. Nevertheless, approximately half of the patients were still considered unfit for a RIT.

## Introduction

Both concurrent chemoradiotherapy (CCRT) and multimodality approaches that include surgery are preferred treatments in fit patients who present with stage III NSCLC.^1^ The PACIFIC trial reported considerable improvements in progression-free^2^ and overall survival^3^ when durvalumab was administered for 12 months after CCRT. Adjuvant durvalumab became standard of care after definitive CCRT.^4^ Nevertheless, the proportion of patients with stage III NSCLC who undergo CCRT can vary considerably between countries and hospitals.^5^^,^^6^

A Dutch population study in patients treated between 2009 and 2013 reported that some form of chemoradiotherapy was administered in 47% of all patients with stage IIIA aged 65 to 74 years and in 20% to 24% of patients aged 75 years and older.^7^ A recent study in our thoracic oncology network revealed that 60% of patients presenting with a stage III NSCLC between 2015 and 2017 underwent chemoradiotherapy, of which 35% was concurrent and 25% sequential.^8^ Patients aged greater than or equal to 70 years and those with a WHO performance score (WHO-PS) greater than or equal to 2 were most likely to not receive a recommendation to undergo either CCRT or surgery combined with other treatment modalities.^8^

We hypothesized that the outcomes of the PACIFIC trial could influence prevailing treatment patterns in The Netherlands. We therefore studied the treatment recommendations made by multidisciplinary tumor boards (MDTs) at three Dutch regional networks between 2015 and 2019 for all patients presenting with a stage III NSCLC. Patterns of use of (adjuvant) durvalumab after 2018 were analyzed.

## Materials and Methods

Three participating regional networks, comprising a total of seven hospitals, collaborated in this study. All institutions maintained written records of MDT recommendations, and all hospitals obtained institutional ethics approval before study participation. At each participating hospital, patients with a stage III NSCLC were identified using data from both the Netherlands Cancer Registry (https://www.iknl.nl/en) and hospital records. All patient details were retrospectively extracted from case records and MDT reports and entered into an ethics-approved database.^8^

Patients who were previously discussed at an MDT outside the three participating regional networks were excluded. For this analysis, CCRT or combined modality approaches that include planned surgery were considered as radical-intent treatment (RIT). RIT definitions used for this analysis were based on the European Society for Medical Oncology guidelines recommending either a surgery-based approach or computed tomography-radiotherapy (RT) for fit patients.^1^^,^^4^ In addition, the efficacy and survival improvements found with adjuvant durvalumab in the PACIFIC study were for patients who had undergone CCRT and not sequential chemoradiotherapy (SCRT). All other treatments were arbitrarily classified as non-RIT (n-RIT) for this analysis, even though the MDT treatment recommendations may have been the most appropriate for the individual patient.

CCRT was defined as having undergone at least two cycles of platinum-based chemotherapy.^4^ Thoracic RT to a total (physical, unadjusted) dose of less than 50 Gy was considered as n-RIT or palliative treatment, as was the receipt of only chemotherapy or other systemic therapies, and no active antitumor therapy (best supportive care). The Charlson comorbidity index (CCI)^9^ was calculated for each patient, and the following comorbidities were recorded: aortic aneurysms, arrhythmias, coronary disease, heart valve disease, hypertension, asthma, any autoimmune disease or immune deficiency, any malignancy 5 years preceding lung cancer diagnosis, and any psychiatric disease. For this study, the CCI malignancy score only included other coexisting malignancies other than stage III NSCLC at baseline. Weight loss was defined as unintentional loss of weight in the 6 months preceding lung cancer diagnosis. All TNM stages and American Joint Committee on Cancer stages were recorded according to the eighth edition.^10^ Adverse events were retrospectively scored according to the Common Terminology Criteria for Adverse Events version 5.0, published on November 27, 2017.

For practical reasons, the date of first MDT presentation was considered the date of diagnosis. For the limited number of patients who were not discussed at an MDT, this date was that of the initial positron emission tomography (or positron emission tomography-computed tomography) scans. Overall survival was calculated from date of diagnosis.

### Statistical Analysis

Patient and tumor characteristics at presentation were compared for differences between regions using the chi-square, Fisher’s exact, and Mann-Whitney *U* tests when appropriate. *p* values less than or equal to 0.05 were considered statistically significant. Univariable logistic regression analysis was performed to identify significant (*p* ≤ 0.05) predictors for recommending RIT. After removing variables with no observed association in univariable analysis (*p* > 0.05), multivariable regression analysis was performed. The forward stepwise selection method was used to identify variables most predictive on the dependent measure. All analyses were carried out using IBM SPSS Statistics for Windows, version 26 (IBM Corp., Armonk, NY).

## Results

**Figure 1:**
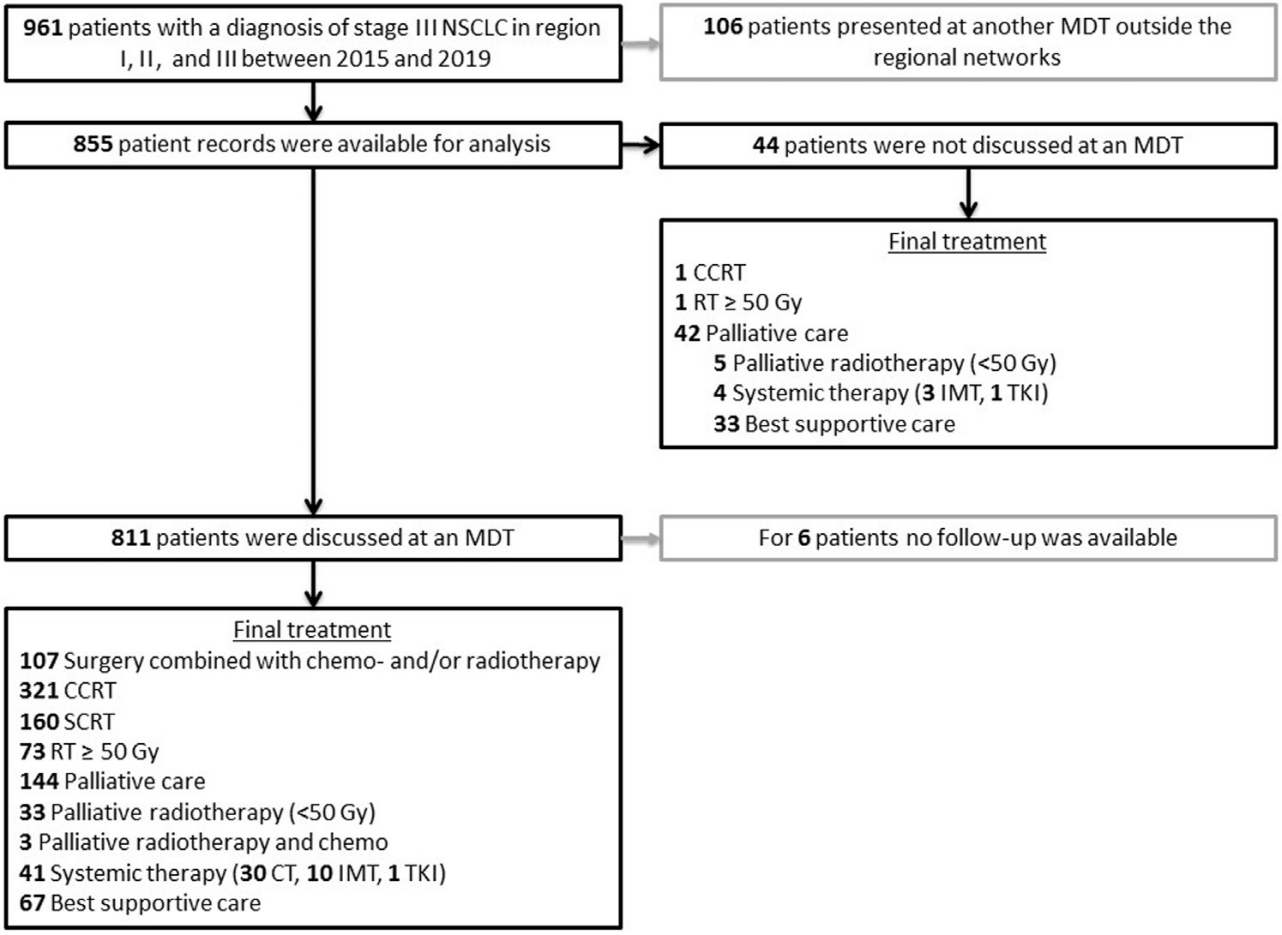
Flow diagram of all patients. Final treatment rates were as follows: in 13% surgery (of which 42% [n = 45] underwent surgery combined with CCRT); 38% CCRT; 19% SCRT; 9% RT greater than or equal to 50 Gy; 22% palliative care; 1% unknown. Thoracic radiotherapy of at least 50 Gy (RT ≥ 50 Gy). CCRT, concurrent chemoradiotherapy; CT, chemotherapy; IMT, immunotherapy; MDT, multidisciplinary tumor board; RT, radiotherapy; SCRT, sequential chemoradiotherapy; TKI, tyrosine kinase inhibitor.

A total of 855 eligible patients were identified, with 475 patients presenting between 2015 and 2017 and 380 between 2018 and 2019 (Fig. 1). Of these, 95% (n = 811) were discussed at an MDT. A total of 106 patients who had been previously discussed at an MDT outside these regional networks were excluded. Patient and tumor characteristics from each region are presented in Table 1. The patients presenting with stage III NSCLC in the three regions had broadly similar characteristics (Supplementary Appendix S1).

**Table 1:** Patient and Tumor Characteristics of All Patients Diagnosed With NSCLC Stage III Between 2015 and 2019 Subdivided Per Regional Network

# *p* ≤ 0.05 between regions within same-time period	Region I			Region II			Region III			All regions		
	2015–2017 (n = 198)	2018–2019 (n = 170)	*p* Value 2015–2017 vs. 2018–2019	2015–2017 (n = 118)	2018–2019 (n = 97)	*p* Value 2015–2017 vs. 2018–2019	2015–2017 (n = 159)	2018–2019 (n = 113)	*p* Value 2015–2017 vs. 2018–2019	2015–2017 (n = 475)	2018–2019 (n = 380)	*p* Value 2015–2017 vs. 2018–2019
Treatment received, n (%)												
Surgery	22 (11)	21 (12)	0.68	12 (10)	17 (18)	0.12	22 (14)	13 (12)	0.55	56 (12)	51 (13)	0.47
CCRT	68 (34)	71 (42)	0.12	45 (38)	42 (43)	0.44	50 (31)	46 (41)	0.13	163 (34)	159 (42)	0.02
SCRT	50 (25)	34 (20)	0.26	17 (14)	10 (10)	0.37	33 (21)	16 (14)	0.15	100 (21)	60 (16)	0.05
RT ≥ 50 Gy	25 (13)	21 (12)	0.97	11 (9)	4 (4)	0.14	10 (6)	3 (3)	0.16	46 (10)	28 (7)	0.23
Palliative care	33 (17)	21 (12)	0.26	32 (27)	23 (24)	0.57	42 (26)	35 (31)	0.45	107 (23)	79 (21)	0.55
Unknown	0 (0)	2 (1)		1 (1)	1 (1)		2 (1)	0 (0)		3 (1)	3 (1)	
Radical-intent therapy, n (%)	90 (45)	92 (54)	0.08	57 (48)	59 (61)	0.06	72 (45)	59 (53)	0.29	219 (46)	210 (55)	0.01
Male, n (%)	107 (54)	101 (59)		77 (65)	58 (60)		76 (48)	72 (64)		260 (55) **#**	231 (61)	0.08
Age, y, median (SD)	69.0 (10.5)	71.0 (9.5)		69.5 (10.3)	71.0 (10.0)		69.0 (10.6)	69.0 (10.4)		69.0 (10.5)	70.0 (9.9)	
Age ≥ 70, y, n (%)	94 (48)	94 (55)	0.14	59 (50)	51 (53)	0.71	73 (46)	53 (47)	0.87	226 (48)	198 (52)	0.19
WHO-PS, n (%)			0.17			0.01			0.09			0.00
0–1	162 (82)	148 (87)		76 (64)	78 (80)		122 (77)	96 (85)		360 (76)	322 (85)	
≥2	31 (16)	15 (9)		27 (23)	14 (14)		26 (16)	12 (11)		84 (18)	41 (11)	
CCI excl. age, mean (SD)	1.37 (1.7)	1.34 (1.6)		1.31 (1.41)	0.98 (1.2)		1.42 (1.7)	1.33 (1.4)		1.37 (1.6)	1.24 (1.5)	
CCI ≥ 2, excl. age, n (%)	56 (33)	62 (31)	0.74	25 (26)	38 (32)	0.30	38 (34)	51 (32)	0.79	119 (31)	151 (32)	0.88
Tumor histology, n (%)												
Adenocarcinoma	88 (44)	74 (44)	0.86	50 (42)	43 (44)	0.77	75 (47)	46 (41)	0.29	213 (45)	163 (43)	0.57
Squamous cell carcinoma	75 (38)	61 (36)	0.69	47 (40)	38 (39)	0.92	55 (35)	38 (34)	0.87	177 (37)	137 (36)	0.72
Other	28 (14)	33 (19)	0.18	5 (4)	10 (10)	0.08	15 (9)	24 (21)	0.01	48 (10)	67 (18)	0.00
None	7 (4)	2 (1)	0.14	16 (14)	6 (6)	0.08	14 (9)	5 (4)	0.16	37 (8)	13 (3)	0.01
AJCC stage, *n* (%)			0.03			0.88			0.36			0.49
IIIA	93 (47)	96 (57)		51 (43)	43 (44)		84 (53)	51 (45)		228 (48)	190 (50)	
IIIB	76 (38)	63 (37)		47 (40)	40 (41)		56 (35)	43 (38)		179 (38)	146 (38)	
IIIC	29 (15)	11 (7)		20 (17)	14 (14)		19 (12)	19 (17)		68 (14)	44 (12)	

AJCC, American Joint Committee on Cancer; CCI, Charlson comorbidity index; CCRT, concurrent chemoradiotherapy; excl., excluding; PS, performance score; RT, radiotherapy; SCRT, sequential chemoradiotherapy.

**Figure 2:**
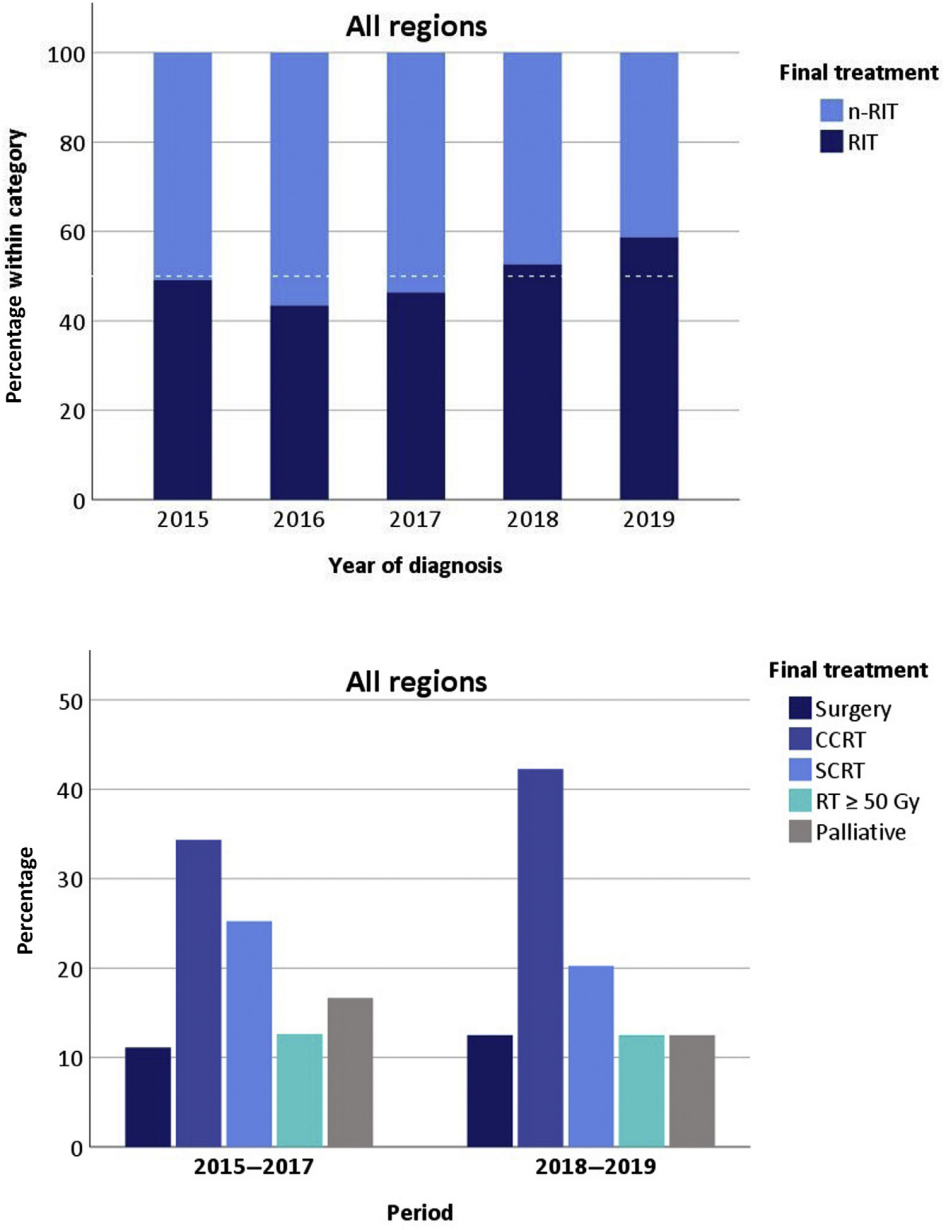
In the period from 2015 to 2017, 46% of patients underwent RITs, and this increased to 55% during the period from 2018 to 2019 (*p* = 0.01), with no differences observed among the three regions (*p* = 0.39). See Supplementary Appendix 1 for an overview of each region. CCRT, concurrent chemoradiotherapy; n-RIT, non–radical-intent treatment; RIT, radical-intent treatment; RT, radiotherapy; SCRT, sequential chemoradiotherapy.

For all 855 patients, the most common reasons stated in MDT or physicians’ notes for not recommending RIT (n = 342) were a poor performance (in 40%, n = 138), patient comorbidities (35%, n = 118), tumor size (14%, n = 47), patient’s preference for not undergoing CCRT or surgery (13%, n = 45), age (11%, n = 36), and unknown reasons (13%, n = 44). The frequencies of RIT and n-RIT finally received by patients per year are illustrated in Figure 2.

**Figure 3:**
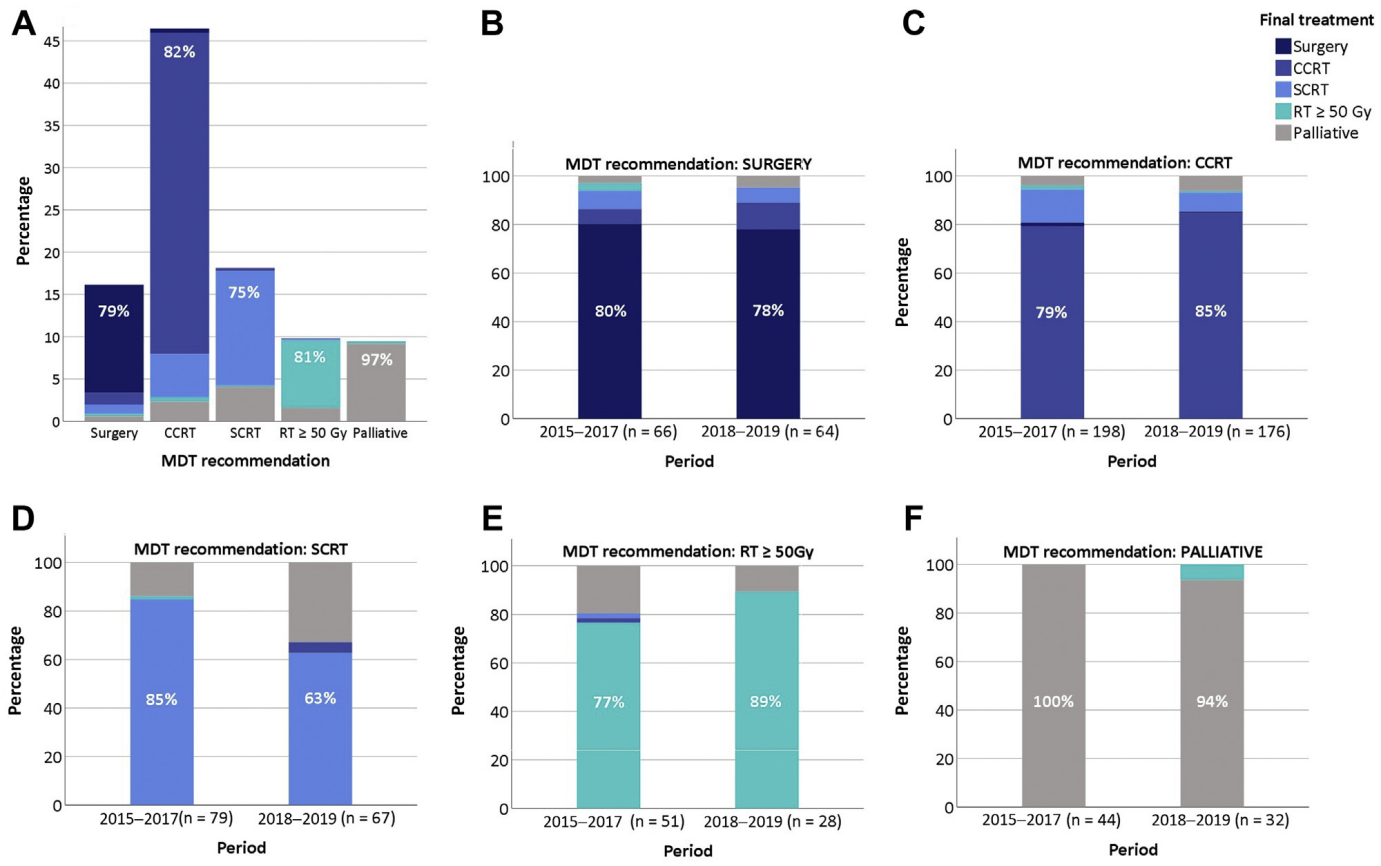
Overview of MDT recommendations versus the actual treatments received by patients with stage III NSCLC. The percentage displayed in the figure represents patients who ultimately received MDT-recommended therapy. Only for those for whom SCRT (D) was the MDT-recommended treatment, a significant difference was observed between the period from 2015 to 2017 and 2018 to 2019, with significantly more switchers to palliative care in the period of 2018 to 2019 (14%–33%, *p* = 0.006). Data on six patients without follow-up data are omitted. CCRT, concurrent chemoradiotherapy; MDT, multidisciplinary tumor board; RT, radiotherapy; SCRT, sequential chemoradiotherapy.

In 811 patients who had been discussed at an MDT, approaches that include surgery were recommended in 16% (n = 132) and CCRT in 47% (n = 378). After the MDT recommendations for a RIT in 63% (n = 510) of patients, only 52% (n = 424) finally underwent RIT consisting of surgery (n = 107) or CCRT (n = 317) (Fig. 3). Post-MDT follow-up details were missing for six patients. No statistically significant differences in recommending RIT were observed between MDT regions (*p* = 0.85).

Of the 80 patients who did not undergo MDT-recommended RIT, most (63%, n = 50) finally underwent SCRT and 37% underwent RT alone to a dose of at least 50 Gy (RT ≥ 50 Gy) *(*n = 6) or palliative care (n = 24). The main reasons for not undergoing MDT-recommended RIT were a poor performance score (29%, n = 24), patient refusal (29%, n = 24), and comorbidity (15%, n = 12). Other reasons included early toxicity (9%, n = 7), tumor size (6%, n = 5), disease progression (6%, n = 5), death (4%, n = 3), a tumor found to be irresectable during surgery (4%, n = 3), age (2%, n = 2), tumor necrosis (2%, n = 2), lack of social support (1%, n = 1), lack of tumor response after initial course of chemotherapy (1%, n = 1), and unknown reasons (4%, n = 2).

On multivariate regression analysis, the factors significantly associated with not undergoing a RIT were the following: (1) age greater than or equal to 70 years; (2) WHO-PS greater than or equal to 2; (3) a CCI greater than or equal to 2 (excluding age); (4) forced expiratory volume in 1 second less than 80% of predicted; (5) nodal stage (N3 disease); and (6) treatment within the period of 2015 to 2017. Table 2 summarizes the factors associated with a failure to undergo RIT.

**Table 2:** Summary of Univariable and Multivariable Logistic Regression Models Predictive for Undergoing RITs From 2015 to 2019

Independent Variables	RIT, n (%)	n-RIT, n (%)	Dependent Variable: Radical-Intent Therapies					
			Univariable			Multivariable		
			OR	95% CI	*p* Value	OR	95% CI	*p* Value
Period, 2015–2019					0.026			0.027
2015–2017	219 (46)	253 (53)	1.00			1.00		
2018–2019	210 (55)	167 (44)	1.45	1.11–1.91		1.53	1.05–2.22	
Age ≥ 70 y	142 (33)	280 (67)	0.25	0.19–0.33	0.000	0.29	0.20–0.42	0.000
WHO-PS ≥ 2	6 (1)	119 (31)	0.03	0.01–0.08	0.000	0.06	0.02–0.17	0.000
CCI value excl. age ≥ 2	90 (21)	179 (43)	0.36	0.26–0.48	0.000	0.55	0.37–0.82	0.004
Weight loss ≤ 6 months before diagnosis	160 (40)	216 (57)	0.51	0.38–0.68	0.000			
FEV1 < 80% of predicted	177 (49)	194 (65)	0.52	0.38–0.71	0.000	0.50	0.34–0.73	0.000
Arrhythmias	43 (10)	62 (15)	0.64	0.43–0.97	0.036			
Autoimmune disease or immune deficiency (≥1)	106 (25)	77 (18)	1.46	1.05–2.03	0.024			
COPD	133 (31)	162 (39)	0.72	0.54–0.95	0.021			
Coronary disease	56 (13)	92 (22)	0.54	0.37–0.77	0.001			
CVA or TIA	32 (8)	69 (16)	0.41	0.26–0.64	0.000			
Dementia	0 (0)	13 (3)	–	–	0.000			
Diabetes mellitus	47 (11)	88 (21)	0.46	0.32–0.68	0.000			
Heart failure	16 (4)	37 (9)	0.40	0.22–0.73	0.003			
Hypertension	122 (28)	155 (37)	0.68	0.51–0.91	0.009			
Moderate to severe chronic kidney disease	10 (2)	37 (9)	0.50	0.35–0.71	0.000			
Other malignancy at time of diagnosis	22 (5)	39 (9)	0.53	0.31–0.91	0.019			
Peripheral vascular disease	31 (7)	48 (11)	0.60	0.38–0.97	0.035			
Comorbidity ≥ 1	330 (77)	379 (90)	0.36	0.24–0.53	0.000			
Adenocarcinoma	223 (52)	150 (36)	1.95	1.48–2.57	0.000			
AJCC stage					0.000			
IIIA	233 (54)	182 (43)	1.00					
IIIB	164 (38)	159 (38)	0.81	0.60–1.08				
IIIC	32 (8)	79 (19)	0.32	0.20–0.50				
cN stage					0.000			
0	73 (17)	40 (10)	1.00			1.00		0.000
1	44 (10)	45 (11)	3.14	1.95–5.05		0.47	0.22–1.02	0.055
2	233 (54)	197 (47)	1.68	1.02–2.77		0.43	0.24–0.78	0.005
3	79 (18)	136 (32)	2.04	1.46–2.85		0.18	0.09–0.35	0.000

AJCC, American Joint Committee on Cancer; CCI, Charlson comorbidity index; CI, confidence interval; COPD, chronic obstructive pulmonary disease; CVA, cerebrovascular accident; excl, excluding; FEV1, forced expiratory volume in 1 second; n-RIT, non–radical-intent treatment; PS, performance score; RIT, radical-intent treatment; TIA, transient ischemic attack.

Age had the largest impact on the likelihood of not undergoing a RIT. Patients aged greater than or equal to 70 years were significantly more likely to have a CCI of greater than or equal to 2 (adjusted for age) (42% versus 22%, *p* = 0.000), a WHO-PS of greater than or equal to 2 (24% versus 7%, *p* = 0.000), squamous cell carcinoma (41% versus 33%, *p* = 0.021), and the following (severe) comorbidities: arrhythmias (17% versus 7%, *p* = 0.000), coronary disease (23% versus 12%, *p* = 0.000), cerebrovascular accident or transient ischemic attack (17% versus 7%, *p* = 0.000), diabetes mellitus (21% versus 11%, *p* = 0.000), heart failure (9% versus 3%, *p* = 0.000), hypertension (42% versus 23%, *p* = 0.000), moderate to severe chronic kidney disease (9% versus 2%, *p* = 0.000), coexisting malignancies (9% versus 5%, *p* = 0.010), and peripheral vascular disease (12% versus 7%, *p* = 0.005).

The proportion of patients who underwent a RIT increased from 46% between 2015 and 2017 to 55% between 2018 and 2019 (*p* = 0.007), and this was due to an increase in the use of CCRT (*p* = 0.023) (Table 2 and Fig. 2). The proportion of patients undergoing CCRT before surgery was similar during both periods (5.1% versus 5.3%, *p* = 0.886). A similar increase in rates of CCRT was observed in all three regions (Supplementary Appendix S2). Despite this increase in CCRT rates, the recorded rates of early toxicity did not reveal an increase (Supplementary Appendix S3). Similarly, mortality rates at 90 days (RIT 1%, SCRT 1%, RT ≥ 50 Gy 3%, palliative 39%) and at 1 year (RIT 19%, SCRT 25%, RT ≥ 50 Gy 44%, palliative 81%) after MDT discussion were constant during both periods (Supplementary Appendix S4). A significant decrease in the proportion of patients undergoing greater than or equal to three cycles of chemotherapy during chemoradiotherapy was observed between periods (72%–60%) with an increase of patients receiving two cycles of chemotherapy (28%–40%) (*p* = 0.005).

Since 2018, a total of 57% of all patients (90 of 159) completing CCRT went on to receive adjuvant durvalumab. This was constant between 2018 and 2019 (57% and 56%, *p* = 0.979). From the patients of this group who had a follow-up of at least 1 year (n = 59), 59% received at least 20 cycles of durvalumab. Reasons recorded for not recommending adjuvant durvalumab were patient refusal (n = 12), performance score (n = 10), progression of disease (n = 10), comorbidity (n = 9), death (n = 8), not recommended in local guidelines (n = 6), programmed death-ligand 1 less than 1% (n = 5), coronavirus disease 2019 pandemic (n = 1), pneumonitis (n = 1), and unknown reasons (n = 10). Eight patients received durvalumab after SCRT. No differences between regions were observed in the rates of durvalumab administration (*p* = 0.237). On logistic regression analysis, factors associated with a failure to undergo adjuvant durvalumab after chemoradiotherapy were an age of greater than or equal to 70 years (*p* = 0.003), diabetes mellitus (*p* = 0.015) and receipt of SCRT (*p* = 0.000) (Table 3 and Supplementary Appendix S5).

**Table 3:** Summary of Univariable and Multivariable Logistic Regression Models Predictive for Receiving Durvalumab After Chemoradiation

Independent Variables	Durva, n (%)	n-Durva, n (%)	Dependent Variable: Durvalumab Treatment After Chemoradiation					
			Univariable			Multivariable		
			OR	95% CI	*p* Value	OR	95% CI	*p* Value
Type					0.000			0.000
Concurrent	90 (92)	69 (57)	1.00			1.00		
Sequential	8 (8)	52 (43)	0.12	0.05–0.27		0.12	0.05–0.30	
Age ≥ 70 y	25 (26)	63 (52)	0.32	0.18–0.56	0.000	0.36	0.18–0.71	0.003
CCI value ≥ 2 (excl. age)	18 (18)	43 (35)	0.41	0.22–0.77	0.005			
Autoimmune disease or immune deficiency (≥1)	47 (48)	37 (31)	2.09	1.20–3.64	0.012			
CVA or TIA	6 (6)	20 (17)	0.33	0.13–0.86	0.021			
Diabetes mellitus	7 (7)	30 (25)	0.23	0.10–0.56	0.000	0.28	0.10–0.78	0.015
Dysphagia grade ≥ 3	5 (5)	17 (14)	0.33	0.12–0.93	0.040	0.27	0.07–1.05	0.058

CCI, Charlson comorbidity index; CI, confidence interval; CVA, cerebrovascular accident; Durva, durvalumab; excl., excluding; n-Durva, non-durvalumab; TIA, transient ischemic attack.

## Discussion

Adjuvant durvalumab therapy after CCRT is associated with improved overall survival in patients with stage III NSCLC.^3^ The rates of CCRT for patients with stage III disease vary considerably in daily practice,^11^, ^12^, ^13^ and the 52% rate of patients undergoing a RIT for stage III NSCLC in this study exceeds those recently reported in England (17%)^13^ and Canada (26%).^12^ MDT evaluation is an essential component of patient care as stage III NSCLC is a heterogeneous disease category, encompassing different primary tumor sizes and extent of nodal metastases. Our study revealed that 95% of all patients with stage III NSCLC at the seven hospitals were discussed at a thoracic MDT, reflecting the current Dutch standard for multidisciplinary lung cancer care.^14^ A RIT was recommended in 63% of all patients, and subsequently, only 52% of patients actually underwent a RIT. The main reported reasons for not undergoing MDT recommended RIT were a deteriorating performance score (29%), patient refusal (29%), and comorbidity (15%).

The high incidence of comorbidities in this patient population highlights the challenges involved in implementing immunotherapy in stage III NSCLC. Full details of comorbidities in 811 patients revealed that independent factors which correlated with a failure to undergo RIT were age greater than or equal to 70 years, WHO-PS greater than or equal to 2, CCI greater than or equal to 2 (age adjusted), forced expiratory volume in 1 second less than 80% of predicted, N3 nodal disease, and treatment in an earlier period (2015–2017). An indication of the potential impact of the PACIFIC study is the significant increase observed in patients undergoing CCRT at all three regions from 2018 to 2019, with a significant decrease of patients undergoing SCRT. As previous reports suggested that increasing use of CCRT can be associated with higher rates of mortality in patients with comorbidity or aged 70 years or older,^15^^,^^16^ it is reassuring that no corresponding increases in 90-day or 1-year mortality were observed in the RIT cohorts in the later periods.

Durvalumab was made available for Dutch patients with nonprogressive stage III NSCLC who had undergone CCRT through an Early Access Program after December 2017. Since September 2019, durvalumab is covered by the Dutch basic health insurance package. Since 2018, a total of 57% of patients in our cohort who had undergone CCRT subsequently received adjuvant durvalumab, a rate which is higher compared with the 43% rate recently reported by a German single center.^17^ Approximately 26% of all patients with stage III disease received immunotherapy in our cohort. In comparison, results from the Dutch Lung Cancer Audit reported that the use of immunotherapy in stage III NSCLC was only 13% in 2018, but increased to 25% in 2019.^18^ This finding could be explained by the fact that only a limited number of Dutch institutions were initially allowed to prescribe durvalumab.^18^ Most centers participating in our study had early access to durvalumab for their patients, but durvalumab was recorded as being unavailable in 9% of eligible patients. Our real-world data are consistent with other reports indicating only between 34% and 73% of patients receive durvalumab after CCRT, with similar reasons being cited for noncompliance.^19^^,^^20^

In September 2018, the European Medicines Agency approved the use of adjuvant durvalumab after completion of CCRT and SCRT, even though the PACIFIC study did not investigate the role of adjuvant durvalumab after SCRT. In our study, only eight patients received durvalumab after completion of SCRT, which was 13% of all SCRT-treated patients after 2018, indicating despite approval of the European Medicines Agency, MDTs continued to follow the PACIFIC schedule.

A notable strength of our analysis is the access to all patient records and the written MDT deliberations for each patient. An important study limitation is the fact that treatment recommendation for stage III NSCLC may vary considerably between Dutch MDTs,^21^ making our findings less representative for Dutch national practice. In addition, full capture of all eligible patients was ensured using both Netherlands Cancer Registry data and hospital records, but we cannot exclude the possibility that some cases of stage III NSCLC might have been missed, such as patients who may not have been staged appropriately or who were not registered in the Netherlands Cancer Registry or MDT notes. Another limitation of this study is that the 1-year survival data for the most recent patient cohorts treated in late 2019 were unavailable. Nevertheless, the timely analysis of data on MDT decision-making and early toxicity remains relevant for identifying area of improvement in patient management so as to minimize gaps between guideline recommendations and real-world practice.

In conclusion, after publication of the PACIFIC study, and with availability of consolidation durvalumab for routine care, an increase in the use of CCRT in stage III NSCLC was observed with rates of early toxicity and mortality being unchanged. Since 2018, most patients undergoing CCRT received durvalumab. Nevertheless, nearly 50% of patients who present with stage III NSCLC were considered unfit to undergo a RIT. The current treatment guidelines are less explicit on how such patients should be managed, and data from ongoing trials evaluating durvalumab after sequential chemotherapy and radiation in less fit patients are awaited (NCT03693300).^22^ Our findings suggest that there is room for MDTs to increase the rate of use of CCRT for stage III NSCLC. The findings also highlight the need for novel clinical trials to address the unmet needs of patients who cannot receive treatment with radical intent, in an era of significant advances in systemic therapies for NSCLC.

## CRediT Authorship Contribution Statement

**Merle I. Ronden:** Methodology, Formal Analysis, Investigation, Resources, Data Curation, Writing—original draft, visualization, project administration.

**Idris Bahce:** Conceptualization, Methodology, Validation, Writing—original draft, supervision, funding acquisition.

**Niels J. M. Claessens, Nicole Barlo:** Resources, Data Curation, Writing—review and editing.

**Max R. Dahele, Johannes M. A. Daniels, Caroline Tissing-Tan, Edo Hekma, Sayed M. S. Hashemi, Antoinet van der Wel, Femke O. B. Spoelstra, Wilko F. A. R. Verbakel, Marian A. Tiemessen, Marjolein van Laren, Annemarie Becker, Svitlana Tarasevych, Cornelis J. A. Haasbeek, Karen Maassen van den Brink, Chris Dickhoff:** Writing—review and editing.

**Suresh Senan:** Conceptualization, Methodology, Validation, Writing—original draft, supervision, funding acquisition.

## Supplementary Data

Supplementary Appendices S1-S5
