# Supplementary material for: The Impact of the Availability of Immunotherapy on Patterns of Care in Stage III NSCLC: A Dutch Multicenter Analysis
Source: JTO Clin Res Rep. 2021 Jun 6;2(7):100195. doi: 10.1016/j.jtocrr.2021.100195 (PMC8474425; doi:10.1016/j.jtocrr.2021.100195)
Supplement: Supplementary Appendices S1-S5 [file mmc1.docx]

|  | **Region I (*n*=366)**  2 missing cases | | | **Region II (*n*=213)**  2 missing cases | | | **Region III (*n*=270)**  2 missing cases | | | **All regions (*n*=849)** | | |
| --- | --- | --- | --- | --- | --- | --- | --- | --- | --- | --- | --- | --- |
|  | **RIT**  **(*n*=182)** | **n-RIT**  **(*n*=184)** | ***p*-value** | **RIT**  **(*n*=116)** | **n-RIT**  **(*n*=97)** | **p-value** | **RIT**  **(*n*=131)** | **n-RIT**  **(*n*=139)** | ***p*-value** | **RIT**  **(*n*=429)** | **n-RIT**  **(*n*=420)** | ***p*-value** |
| **Presented at an MDT, *n* (%)** | 182 (100%) | 174 (95%) | **0.00** | 116 (100%) | 75 (77%) | **0.00** | 130 (99%) | 128 (92%) | **0.00** | 428 (99%) | 377 (90%) | **0.00** |
| **Male, *n* (%)** | 103 (57%) | 105 (57%) | 0.93 | 68 (59%) | 66 (68%) | 0.16 | 74 (57%) | 74 (53%) | 0.59 | 245 (57%) | 245 (58%) | 0.72 |
| **Age, years, median (SD)** | 65.5 (9.0) | 74.0 (9.5) |  | 66.0 (9.2) | 74.0 (9.7) |  | 65.0 (9.7) | 72.0 (9.7) |  | 66.0 (9.3) | 74.0 (9.6) |  |
| **Age (years), *n* (%)** |  |  | **0.00** |  |  | **0.00** |  |  | **0.00** |  |  | **0.00** |
| <50 | 8 (4%) | 4 (2%) |  | 6 (5%) | 2 (2%) |  | 14 (11%) | 2 (1%) |  | 28 (7%) | 8 (2%) |  |
| 50-59 | 44 (24%) | 17 (9%) |  | 24 (21%) | 4 (4%) |  | 27 (21%) | 17 (12%) |  | 95 (22%) | 38 (9%) |  |
| 60-69 | 73 (40%) | 33 (18%) |  | 42 (36%) | 26 (27%) |  | 49 (37%) | 35 (25%) |  | 164 (38%) | 94 (22%) |  |
| 70-79 | 55 (30%) | 90 (49%) |  | 41 (35%) | 34 (35%) |  | 36 (28%) | 47 (34%) |  | 132 (31%) | 171 (41%) |  |
| ≥ 80 | 2 (1%) | 40 (22%) |  | 3 (3%) | 31 (32%) |  | 5 (4%) | 38 (27%) |  | 10 (2%) | 109 (26%) |  |
| **Age ≥70 years, *n* (%)** | 57 (31%) | 130 (71%) | **0.00** | 44 (38%) | 65 (67%) | **0.00** | 41 (31%) | 85 (61%) | **0.00** | 142 (33%) | 280 (67%) | **0.00** |
| **WHO-PS, *n* (%)** |  |  | **0.00** |  |  | **0.00** |  |  | **0.00** |  |  | **0.00** |
| 0-1 | 179 (98%) | 130 (71%) |  | 105 (91%) | 47 (49%) |  | 128 (98%) | 89 (64%) |  | 412 (96%) | 266 (63%) |  |
| ≥2 | 2 (1%) | 44 (24%) |  | 4 (3%) | 37 (38%) |  | 0 (0%) | 38 (27%) |  | 6 (1%) | 119 (28%) |  |
| **CCI value, *n* (%)** |  |  | **0.00** |  |  | **0.00** |  |  | **0.00** |  |  | **0.00** |
| 0-1 | 30 (17%) | 8 (4%) |  | 19 (16%) | 5 (5%) |  | 28 (21%) | 6 (4%) |  | 77 (18%) | 19 (5%) |  |
| 2-4 | 127 (70%) | 94 (51%) |  | 78 (67%) | 49 (51%) |  | 86 (66%) | 71 (51%) |  | 291 (68%) | 215 (51%) |  |
| ≥5 | 25 (14%) | 82 (45%) |  | 19 (16%) | 5 (5%) |  | 17 (13%) | 62 (45%) |  | 61 (14%) | 187 (44%) |  |
| **CCI value excl age, *n* (%)** |  |  | **0.00** |  |  | **0.00** |  |  | **0.00** |  |  | **0.00** |
| 0-1 | 143 (79%) | 105 (57%) |  | 93 (80%) | 57 (59%) |  | 103 (79%) | 79 (57%) |  | 339 (79%) | 241 (57%) |  |
| 2-4 | 33 (18%) | 64 (35%) |  | 20 (17%) | 36 (37%) |  | 26 (20%) | 46 (33%) |  | 79 (18%) | 146 (35%) |  |
| ≥5 | 6 (3%) | 15 (8%) |  | 3 (3%) | 4 (4%) |  | 2 (2%) | 14 (10%) |  | 11 (3%) | 33 (8%) |  |
| **Weight loss <6 months prior to diagnosis, *n* (%)** | 74 (41%) | 97 (53%) | **0.04** | 38 (33%) | 51 (53%) | **0.00** | 48 (37%) | 68 (49%) | 0.11 | 160 (37%) | 216 (51%) | **0.00** |
| **Smoking history, *n* (%)** | 174 (96%) | 178 (97%) | 0.24 | 111 (96%) | 87 (90%) | 0.77 | 123 (94%) | 134 (96%) | 0.48 | 408 (95%) | 399 (95%) | 0.31 |
| **FEV1<80% of predicted, *n* (%)** | 71 (39%) | 89 (48%) | **0.00** | 37 (32%) | 29 (30%) | **0.03** | 69 (53%) | 76 (55%) | 0.21 | 177 (41%) | 194 (46%) | **0.00** |
| **Medical History, *n* (%)** |  |  |  |  |  |  |  |  |  |  |  |  |
| Arrhythmias | 14 (8%) | 21 (11%) | 0.23 | 11 (10%) | 16 (17%) | 0.13 | 18 (14%) | 25 (18%) | 0.34 | 43 (10%) | 62 (15%) | **0.04** |
| Asthma | 15 (8%) | 6 (3%) | **0.04** | 5 (5%) | 10 (10%) | 0.16 | 7 (5%) | 8 (6%) | 0.88 | 28 (7%) | 24 (6%) | 0.62 |
| Autoimmune disease or immune deficiency (≥1) | 50 (28%) | 35 (19%) | 0.06 | 30 (26%) | 16 (17%) | 0.10 | 26 (20%) | 26 (19%) | 0.81 | 106 (25%) | 77 (18%) | **0.02** |
| COPD | 55 (30%) | 74 (40%) | **0.05** | 35 (30%) | 30 (31%) | 0.91 | 43 (33%) | 58 (42%) | 0.13 | 133 (31%) | 162 (39%) | **0.02** |
| Coronary disease | 20 (11%) | 42 (23%) | **0.00** | 16 (14%) | 19 (20%) | 0.26 | 20 (15%) | 31 (22%) | 0.14 | 56 (13%) | 92 (22%) | **0.00** |
| CVA or TIA | 14 (8%) | 25 (14%) | 0.07 | 9 (8%) | 21 (22%) | **0.00** | 9 (7%) | 23 (17%) | **0.01** | 32 (8%) | 69 (17%) | **0.00** |
| Dementia | 0 (0%) | 4 (2%) | 0.12 | 0 (0%) | 6 (6%) | **0.01** | 0 (0%) | 4 (3%) | 0.12 | 0 (0%) | 14 (3%) | **0.00** |
| Diabetes mellitus | 18 (10%) | 41 (22%) | **0.00** | 13 (11%) | 22 (23%) | **0.03** | 16 (12%) | 25 (18%) | 0.19 | 47 (11%) | 88 (21%) | **0.00** |
| Heart failure | 5 (3%) | 13 (7%) | 0.06 | 7 (6%) | 12 (12%) | 0.15 | 4 (3%) | 12 (9%) | 0.07 | 16 (4%) | 37 (9%) | **0.00** |
| Heart valve disease | 10 (6%) | 9 (5%) | 0.80 | 7 (6%) | 6 (6%) | 0.96 | 8 (6%) | 8 (6%) | 0.90 | 25 (6%) | 23 (6%) | 0.83 |
| Hemiplegia | 0 (0%) | 2 (1%) | 0.16 | 0 (0%) | 0 (0%) | 1.00 | 0 (0%) | 1 (1%) | 1.00 | 0 (0%) | 3 (1%) | 0.12 |
| Hypertension | 44 (24%) | 70 (38%) | **0.00** | 36 (31%) | 34 (35%) | 0.53 | 42 (32%) | 51 (37%) | 0.42 | 122 (28%) | 155 (37%) | **0.00** |
| Moderate to severe chronic kidney disease | 2 (1%) | 16 (9%) | **0.00** | 4 (3%) | 11 (11%) | **0.03** | 4 (3%) | 10 (7%) | 0.13 | 10 (2%) | 37 (9%) | **0.00** |
| Other malignancy at time of diagnosis | 15 (8%) | 18 (10%) | 0.72 | 2 (2%) | 4 (4%) | 0.42 | 5 (4%) | 17 (12%) | **0.01** | 22 (5%) | 39 (9%) | **0.02** |
| Peripheral vascular disease | 13 (7%) | 21 (11%) | 0.16 | 10 (9%) | 9 (9%) | 1.00 | 8 (6%) | 18 (13%) | 0.07 | 31 (7%) | 48 (11%) | **0.44** |
| Psychiatric disease | 7 (4%) | 15 (8%) | 0.12 | 8 (7%) | 9 (9%) | 0.62 | 7 (5%) | 10 (7%) | 0.62 | 22 (5%) | 34 (8%) | 0.10 |
| Comorbidity ≥1 | 138 (76%) | 161 (88%) | **0.00** | 93 (80%) | 89 (92%) | **0.02** | 99 (76%) | 129 (93%) | **0.00** | 330 (77%) | 379 (90%) | **0.00** |
| **Tumor Histology, *n* (%)** |  |  |  |  |  |  |  |  |  |  |  |  |
| Adenocarcinoma | 93 (51%) | 68 (37%) | **0.01** | 62 (53%) | 30 (31%) | **0.00** | 68 (52%) | 52 (37%) | **0.02** | 223 (52%) | 150 (36%) | **0.00** |
| Squamous cell carcinoma | 55 (30%) | 80 (44%) | **0.01** | 46 (40%) | 38 (39%) | 0.943 | 46 (35%) | 47 (34%) | 0.822 | 147 (34%) | 165 (39%) | 0.13 |
| Other | 33 (18%) | 28 (15%) | 0.45 | 8 (7%) | 7 (7%) | 0.93 | 16 (12%) | 22 (16%) | 0.39 | 57 (13%) | 57 (14%) | 0.90 |
| None | 1 (1%) | 8 (4%) | **0.02** | 0 (0%) | 22 (23%) | **0.00** | 1 (1%) | 18 (13%) | **0.00** | 2 (1%) | 48 (11%) | **0.00** |
| **AJCC stage, *n* (%)** |  |  | **0.03** |  |  | **0.00** |  |  | **0.01** |  |  | **0.00** |
| IIIA | 99 (54%) | 89 (48%) |  | 60 (52%) | 34 (35%) |  | 74 (57%) | 59 (42%) |  | 233 (54%) | 182 (43%) |  |
| IIIB | 71 (39%) | 67 (36%) |  | 47 (41%) | 39 (40%) |  | 46 (35%) | 53 (38%) |  | 164 (38%) | 159 (38%) |  |
| IIIC | 12 (7%) | 28 (15%) |  | 9 (8%) | 24 (25%) |  | 11 (8%) | 27 (19%) |  | 32 (8%) | 79 (19%) |  |
| **cT-stage, *n* (%)** |  |  | 0.23 |  |  | 0.21 |  |  | 0.53 |  |  | 0.13 |
| 1-2 | 69 (38%) | 66 (36%) |  | 43 (37%) | 25 (26%) |  | 41 (31%) | 35 (25%) |  | 153 (36%) | 126 (30%) |  |
| 3-4 | 108 (59%) | 106 (58%) |  | 71 (61%) | 70 (72%) |  | 77 (59%) | 90 (65%) |  | 256 (60%) | 266 (63%) |  |
| x | 5 (3%) | 12 (7%) |  | 2 (2%) | 2 (2%) |  | 13 (10%) | 14 (10%) |  | 20 (5%) | 28 (7%) |  |
| **cN-stage, *n* (%)** |  |  | **0.11** |  |  | **0.02** |  |  | **0.00** |  |  | **0.00** |
| 0 | 30 (17%) | 20 (11%) |  | 19 (16%) | 9 (9%) |  | 24 (18%) | 11 (8%) |  | 73 (17%) | 40 (10%) |  |
| 1 | 12 (7%) | 14 (8%) |  | 13 (11%) | 8 (8%) |  | 19 (15%) | 23 (17%) |  | 44 (10%) | 45 (11%) |  |
| 2 | 106 (58%) | 98 (53%) |  | 63 (54%) | 45 (46%) |  | 64 (49%) | 54 (39%) |  | 233 (54%) | 197 (47%) |  |
| 3 | 34 (19%) | 50 (27%) |  | 21 (18%) | 35 (36%) |  | 24 (18%) | 51 (37%) |  | 79 (18%) | 136 (32%) |  |

**S1.** Patient characteristics subdivided in RIT and n-RIT per region 2015-2019. Within the RIT group, no statistical differences were observed between regions.


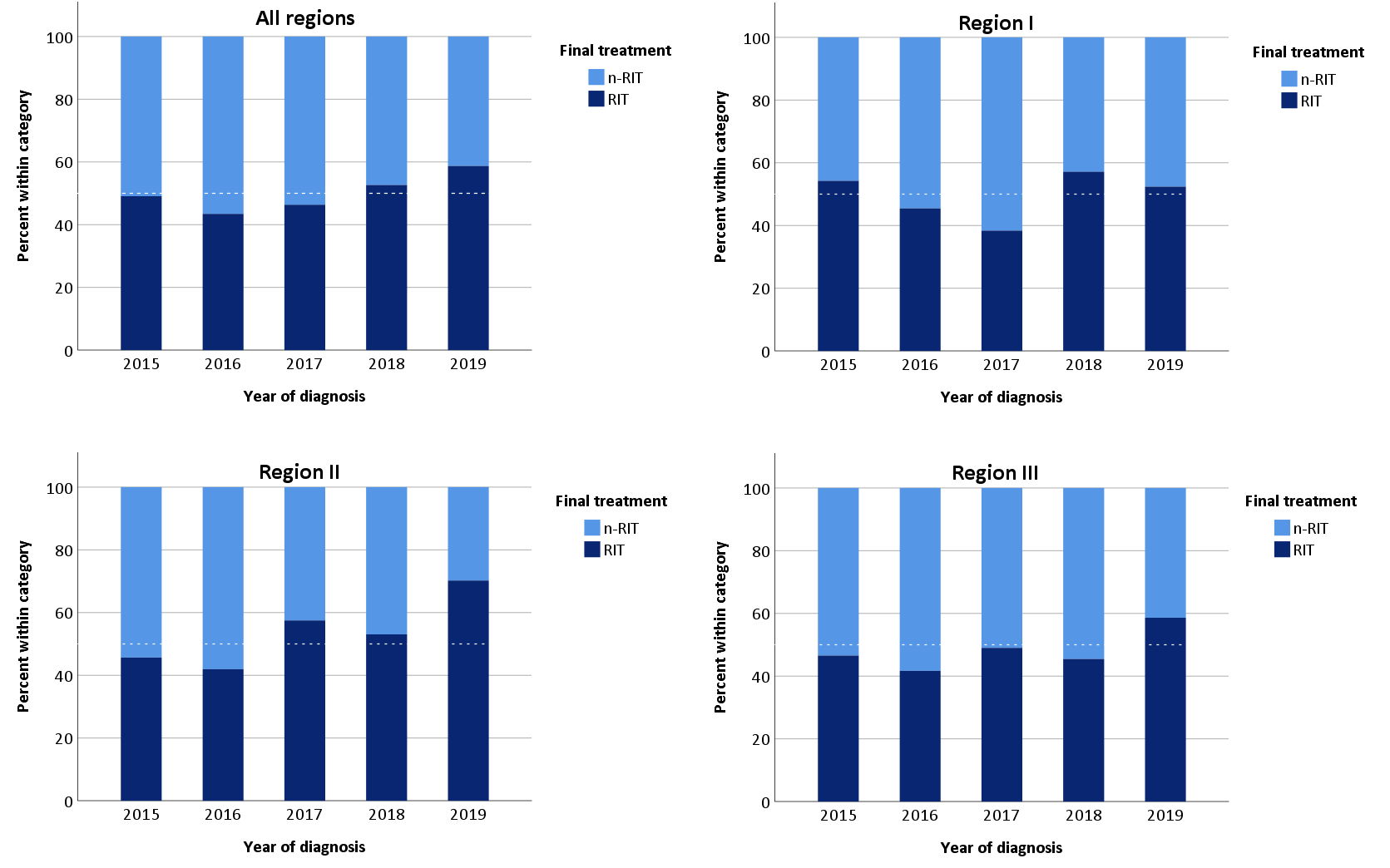


**S2.** Overview of the distribution of RIT and n-RIT treatments per region per year. RIT: radical intent treatments; nRIT: non-radical intent treatments**.**

| **Final treatment** | **Surgery**  **(*n*=107)** | | | p-value ’15-’17 vs. ’18-‘19 | **CCRT**  **(*n*=322)** | | | p-value ’15-’17 vs. ’18-‘19 | **SCRT**  **(*n*=160)** | | | p-value ’15-’17 vs. ’18-‘19 | **RT**≥**50Gy**  **(*n*=74)** | | | p-value ’15-’17 vs. ’18-‘19 |
| --- | --- | --- | --- | --- | --- | --- | --- | --- | --- | --- | --- | --- | --- | --- | --- | --- |
|  | ’15-‘17  (*n*=56) | ’18-‘19  (*n*=51) | |  | ’15-‘17  (*n*=163) | ’18-‘19  (*n*=159) | |  | ’15-‘17  (*n*=100) | ’18-‘19  (*n*=60) | |  | ’15-‘17  (*n*=46) | ’18-‘19  (*n*=28) | |  |
| **Pneumonitis, *n* (%)** |  |  | |  |  |  | |  |  |  | |  |  |  | |  |
| Grade ≥2 | 3 (5.4%) | | 0 (0%) | 0.25 | 29 (18%) | | 26 (16%) | 0.77 | 17 (17%) | | 8 (13%) | 0.66 | 3 (6.5%) | | 0 (0%) | 0.29 |
| Grade ≥3 | 1 (1.8%) | | 0 (0%) | 1.00 | 3 (1.9%) | | 6 (3.8%) | 0.33 | 2 (2.0%) | | 2 (3.3%) | 0.63 | 1 (2.2%) | | 0 (0%) | 1.00 |
| **Dysphagia, *n* (%)** |  | |  |  |  | |  |  |  | |  |  |  | |  |  |
| Grade ≥2 | 8 (14%) | | 2 (3.9%) | 0.10 | 61 (38%) | | 53 (33%) | 0.48 | 27 (27%) | | 10 (17%) | 0.18 | 13 (28%) | | 1 (3.6%) | **0.01** |
| Grade ≥3 | 1 (1.8%) | | 0 (0%) | 1.00 | 23 (14%) | | 17 (11%) | 0.40 | 7 (7.0%) | | 5 (8.3%) | 0.76 | 2 (4.3%) | | 0 (0%) | 0.52 |
| **Other toxicity, *n* (%)** |  | |  |  |  | |  |  |  | |  |  |  | |  |  |
| Grade ≥3 | 9 (16%) | | 6 (12%) | 0.59 | 22 (14%) | | 19 (12%) | 0.56 | 16 (16%) | | 7 (12%) | 0.50 | 1 (2.2%) | | 1 (3.6%) | 1.00 |

**S3.** Overview of early toxicity in patients with stage III NSCLC in the period of 2015-2019.

| **Final treatment** | **Surgery** | | ***p*-value ’15-’17 vs. ’18-‘19** | **CCRT** | | ***p*-value ’15-’17 vs. ’18-‘19** | **SCRT** | | ***p*-value ’15-’17 vs. ’18-‘19** | **RT**≥**50Gy** | | ***p*-value ’15-’17 vs. ’18-‘19** | **Palliative** | | ***p*-value ’15-’17 vs. ’18-‘19** |
| --- | --- | --- | --- | --- | --- | --- | --- | --- | --- | --- | --- | --- | --- | --- | --- |
| **30-day mortality** | **0%** | 0/107 | **-** | **0%** | 0/322 | **-** | **0%** | 0/160 | **-** | **0%** | 0/74 | **-** | **16%** | 29/182 | 0.22 |
| 2015-2017 | 0% | 0/56 |  | 0% | 0/163 |  | 0% | 0/100 |  | 0% | 0/46 |  | 19% | 20/105 |  |
| 2018-2019 | 0% | 0/51 |  | 0% | 0/159 |  | 0% | 0/60 |  | 0% | 0/28 |  | 12% | 9/77 |  |
| **90-day mortality** | **0%** | 0/107 | **-** | **2%** | 6/322 | 0.44 | **1%** | 1/160 | 1.00 | **3%** | 2/74 | 0.52 | **39%** | 71/182 | 0.22 |
| 2015-2017 | 0% | 0/56 |  | 1% | 2/163 |  | 1% | 1/100 |  | 4% | 2/46 |  | 43% | 45/105 |  |
| 2018-2019 | 0% | 0/51 |  | 3% | 4/159 |  | 0% | 0/60 |  | 0% | 0/28 |  | 34% | 26/77 |  |
| **180-day mortality** | **3%** | 3/107 | 0.25 | **7%** | 21/322 | 0.51 | **9%** | 14/160 | 0.25 | **18%** | 13/74 | 0.54 | **61%** | 109/180 | 0.88 |
| 2015-2017 | 5% | 3/56 |  | 6% | 9/163 |  | 11% | 11/100 |  | 15% | 7/46 |  | 60% | 63/105 |  |
| 2018-2019 | 0% | 0/51 |  | 8% | 12/159 |  | 5% | 3/60 |  | 21% | 6/28 |  | 61% | 46/75 |  |
| **1-year mortality** | **14%** | 14/101 | 0.57 | **20%** | 61/303 | 0.76 | **25%** | 38/155 | 0.70 | **44%** | 31/71 | 0.14 | **81%** | 141/175 | 0.57 |
| 2015-2017 | 16% | 9/55 |  | 21% | 34/163 |  | 26% | 26/100 |  | 37% | 17/46 |  | 79% | 83/105 |  |
| 2018-2019 | 11% | 5/46 |  | 19% | 27/140 |  | 22% | 12/55 |  | 56% | 14/25 |  | 83% | 58/70 |  |

**S4.** Overview of early mortality in patients with stage III NSCLC in the period of 2015-2019. Missing cases: 30-, 90-day mortality: 10, 180-day mortality: 12, 1-year mortality: 50.

**S5.** Characteristics of patients who received chemoradiotherapy, either concurrent or sequential from 2018.

|  | **Durvalumab (*n*=98)** | **No durvalumab**  **(*n*=121)** | ***p*-value** |
| --- | --- | --- | --- |
| **Treatment type, *n* (%)** |  |  | **0.00** |
| Concurrent chemoradiotherapy | 90 (92%) | 69 (75%) |  |
| Sequential chemoradiotherapy | 8 (8%) | 52 (43%) |  |
| **Male, n (%)** | 55 (56%) | 69 (57%) | 0.89 |
| **Age, years, median (SD)** | 64.0 (7.6) | 70.0 (8.4) |  |
| **Age (years), *n* (%)** |  |  | **0.00** |
| <50 | 2 (2%) | 4 (3%) |  |
| 50-59 | 31 (32%) | 15 (12%) |  |
| 60-69 | 40 (41%) | 39 (32%) |  |
| 70-79 | 22 (22%) | 59 (49%) |  |
| ≥ 80 | 3 (3%) | 4 (3%) |  |
| **Age ≥70 years, *n* (%)** | 25 (26%) | 63 (52%) | **0.00** |
| **WHO-PS, *n* (%)** |  |  | 0.25 |
| 0-1 | 97 (100%) | 11 (97%) |  |
| ≥2 | 0 (0%) | 3 (3%) |  |
| **CCI value ≥2 (excl age), *n* (%)** | 18 (18%) | 43 (35%) | **0.05** |
| **Weight loss <6 months prior to diagnosis, *n* (%)** | 39 (40%) | 51 (42%) | 0.75 |
| **FEV1<80% of predicted, *n* (%)** | 43 (44%) | 56 (46%) | 0.52 |
| **Medical History, *n* (%)** |  |  |  |
| Arrhythmias | 6 (6%) | 14 (12%) | 0.24 |
| Asthma | 5 (5%) | 11 (9%) | 0.31 |
| Autoimmune disease or immune deficiency (≥1) | 47 (48%) | 37 (31%) | **0.01** |
| COPD | 36 (37%) | 43 (36%) | 0.89 |
| Coronary disease | 11 (11%) | 23 (19%) | 0.14 |
| CVA or TIA | 6 (6%) | 20 (17%) | **0.02** |
| Diabetes mellitus | 7 (7%) | 30 (25%) | **0.00** |
| Heart failure | 5 (5%) | 7 (6%) | 1.00 |
| Heart valve disease | 8 (8%) | 8 (7%) | 0.80 |
| Hypertension | 29 (30%) | 48 (40%) | 0.16 |
| Moderate to severe chronic kidney disease | 1 (1%) | 3 (3%) | 0.63 |
| Other malignancy at time of diagnosis | 5 (5%) | 11 (9%) | 0.31 |
| Peripheral vascular disease | 7 (7%) | 10 (8%) | 0.81 |
| Psychiatric disease | 4 (4%) | 6 (5%) | 1.00 |
| Comorbidity ≥1 | 76 (78%) | 103 (85%) | 0.16 |
| **Tumor Histology*, n (%)*** |  |  | 0.37 |
| Adenocarcinoma | 50 (51%) | 51 (42%) |  |
| Squamous cell carcinoma | 30 (31%) | 47 (39%) |  |
| Other | 18 (18%) | 23 (19%) |  |
| **AJCC stage*, n (%)*** |  |  | 0.45 |
| IIIA | 46 (47%) | 57 (47%) |  |
| IIIB | 39 (40%) | 54 (45%) |  |
| IIIC | 13 (13%) | 10 (8%) |  |
| **cT-stage*, n (%)*** |  |  | 0.47 |
| 1-2 | 39 (40%) | 39 (32%) |  |
| 3-4 | 52 (53%) | 74 (61%) |  |
| x | 7 (7%) | 8 (7%) |  |
| **cN-stage, *n* (%)** |  |  | 0.47 |
| 0 | 11 (11%) | 12 (10%) |  |
| 1 | 5 (5%) | 13 (11%) |  |
| 2 | 59 (60%) | 71 (59%) |  |
| 3 | 23 (24%) | 24 (20%) |  |
| **Pneumonitis grade ≥2, *n* (%)** | 17 (17%) | 17 (14%) | 0.58 |
| **Pneumonitis grade ≥3, *n* (%)** | 3 (3%) | 5 (4%) | 0.73 |
| **Dysphagia grade ≥2, *n* (%)** | 25 (26%) | 38 (31%) | 0.37 |
| **Dysphagia grade ≥3, *n* (%)** | 5 (5%) | 17 (14%) | **0.04** |
